# Effect of Corn Dried Distiller Grains with Solubles (DDGS) in Dairy Cow Diets on Manure Bioenergy Production Potential

**DOI:** 10.3390/ani4010082

**Published:** 2014-03-05

**Authors:** Daniel I. Massé, Guillaume Jarret, Chaouki Benchaar, Noori M. Cata Saady

**Affiliations:** Agriculture and Agri-Food Canada, 2000 College Street, Sherbrooke, Quebec, J1M 0C8, Canada; E-Mails: Guillaume.Jarret@agr.gc.ca (G.J.); Chaouki.Benchaar@agr.gc.ca (C.B.); Noori.Saady@agr.gc.ca (N.M.C.S.)

**Keywords:** corn DDGS, dairy, manure, methane, bioenergy, psychrophilic

## Abstract

**Simple Summary:**

Among the measures proposed to reduce environmental pollution from the livestock sector, animal nutrition has a strong potential to reduce enteric and manure storages methane emissions. Changes in diet composition also affect the bioenergy potential of dairy manures. Corn dried distillers grains with solubles (DDGS), which are rich in fat, can be included in animal diets to reduce enteric methane (CH_4_) emissions, while increasing the bioenergy potential of the animal manure during anaerobic digestion. The inclusion of 30% DDGS in the cow diet caused a significant increase of 14% in daily bioenergy production (_N_L methane day^−1^·cow^−1^).

**abstract:**

The main objective of this study was to obtain scientifically sound data on the bioenergy potential of dairy manures from cows fed different levels of corn dried distillers grains with solubles (DDGS). Three diets differing in corn DDGS content were formulated: 0% corn DDGS (DDGS0; control diet), 10% corn DDGS (DDGS10) and 30% corn DDGS (DDGS30). Bioenergy production was determined in psychrophilic (25 ± 1 °C) sequencing batch reactors (SBRs) fed 3 g COD L^−1^·day^−1^ during a two-week feeding period followed by a two-week react period. Compared to the control diet, adding DDGS10 and DDGS30 to the dairy cow diet increased the daily amount of fat excreted in slurry by 29% and 70%, respectively. The addition of DDGS30 increased the cows’ daily production of fresh feces and slurry by 15% and 11%, respectively. Furthermore, the incorporation of DDGS30 in the diet increased the daily amounts of dry matter (DM), volatile solids (VS), neutral detergent fiber (NDF), acid detergent fiber (ADF) and hemicellulose by 18%, 18%, 30%, 15% and 53%, respectively, compared to the control diet. While the addition of DDGS did not significantly affect the specific CH_4_ production per kg VS compared to the control diet, DDGS30 increased the per cow daily CH_4_ production by 14% compared to the control diet.

## 1. Introduction

Manure produced by livestock operations contributes to greenhouse gas (GHG) emissions. In Canada, GHG emissions produced by the livestock sector represent about 8% of total national GHG emissions, and manure accounts for about 12% of agricultural emissions [1]. Methane (CH_4_), one of the principal agricultural GHGs, is produced by enteric fermentation in ruminant animals [2] and by anaerobic fermentation of manure in livestock buildings and manure storage facilities [3,4]. The level of CH_4_ emissions during manure storage is affected by environmental factors such as storage temperature [5,6], storage duration [7], manure composition, and bedding content [7,8,9]. Environmental legislation and public concern about the environmental footprint of livestock production operations have increased the pressure on producers to take measures to reduce atmospheric and environmental pollution. Among all the measures proposed to reduce environmental pollution from the livestock sector, animal nutrition has a strong potential to reduce enteric and manure storages CH_4_ emissions. Changes in diet composition may also affect the bioenergy potential of dairy manures. The recent increase in biofuel by-products, such as corn dried distillers grains with solubles (DDGS), which are rich in fat, may be a good candidate by-product to include in animal diets with a view to reducing enteric CH_4_ emissions [10]. The addition of fat to a diet reduces or eliminates protozoa as well as methanogenic bacteria in the rumen, resulting in decreased CH_4_ emissions and a shift in the hydrogen sink through bio-hydrogenation via propionate production [11,12]. DDGS by-products are also rich in fiber complexes with nutrients (such as protein and carbohydrates) that are partially digested in the rumen and gut of the animal. This could increase the amounts of nutrients available for microbial fermentation in the slurry, thus potentially facilitating a compensatory increase of CH_4_ production as reported in the study of Külling *et al.* [13]. Jarret *et al.* [14] showed that the inclusion of wheat DDGS in pig diets could modify manure quantity and characteristics and thus alter the GHG budget of manure during anaerobic digestion by increasing biogas production. Green energy recovery via biogas production combined with on-farm power/heat generation seems the most logical approach for reducing fossil fuel use at the farm level and the carbon footprint of dairy products. This study used an integrated approach to assess manure-based bioenergy recovery potential in relation to dairy cow diets compositions. Within this context, the objective of this study was to investigate the effect DDGS level in the dairy cow diet on manure bioenergy production. 

## 2. Materials and Methods

### 2.1. Experimental Design

As part of an integrated research project to assess the carbon footprint of milk products in Canada, two experiments were conducted: (1) An animal experiment to evaluate the impact of the level of corn DDGS inclusion as a fat source in Holstein cow’s diets on enteric CH_4_ emissions and on milk yield and composition; (2) A bioenergy potential assessment experiment.

In the animal experiment (used Latin square design), diets containing four different levels of steam-flaked corn DDGS (dry matter-based): 0% - DDGS0, considered as the control diet; 10% - DDGS10; 20% - DDGS20; and 30% - DDGS30 was fed to 16 lactating Holstein cows (645 ± 49 kg) were fed (*i.e.*, 4 diets × 4 cows involved for each diet = 16 cows in each experimental period); four testing periods (duration of each testing period was 4 months) were conducted as per the Latin square design. For the assessment of the effect of diet composition on manure physico-chemical characteristics, urine and feces were collected daily from the 16 cows involved in the 4 diet testing periods. 

The collection of feces and urine has been described elsewhere [15]. Briefly, Cows were fitted with harnesses and tubes allowing the collection of feces and urine separately. Feces were weighed and mixed daily, and a representative sample (2%) was collected, stored at −20 °C, and subsequently thawed, freeze dried, and ground to pass a 1-mm screen using a Wiley mill for later analysis of DM, VS, total N, NDF, ADF and other parameters. Total urine was collected daily into reinforced plastic containers. The composition of the diets is presented in Table 1 [15].

In the bioenergy potential assessment experiment, the manure (cow feces and urine) produced from the DDGS0, DDGS10, DDGS30 diets during the first testing period only has been used as substrate for anaerobic digestion. Therefore, for the bioenergy potential assessment experiment 12 cows were involved (4 cows for each diet × 3 diets (DDGS0, DDGS10, DDGS30)). The manure slurries were stored in 200-L containers maintained at 4 °C. At the end of the collection period, the slurries were homogenized using a portable mechanical mixer and subsamples were taken for analysis.

**Table 1:** Ingredients and composition of the three diets tested: 0, 10% and 30% dried distillers grains with solubles (DDGS0, DDGS10 and DDGS30), and volume and composition of dairy cow slurry (kg·day^−1^·cow^−1^) as a function of feeding strategy.

	DDGS0	DDGS10	DDGS30	SEM^α^	*p*-Value
**Ingredients, % DM^β^**					
Alfalfa silage	22.9	22.9	22.9		
Corn silage	33.8	33.8	33.8		
Timothy hay	3.4	3.4	3.4		
Steam-flaked corn	16.7	11.0	0.0		
Soybean meal	13.2	8.8	0.0		
Corn dried distillers grains with Solubles	0.0	10.1	30.0		
Beet pulp, dehydrated	7.6	7.6	7.5		
Calcium carbonate	0.7	0.7	0.8		
Mineral and vitamin supplement	1.6	1.6	1.5		
**Composition, % DM**					
Organic matter	93.0	92.9	92.5		
Crude protein	16.2	16.4	16.8		
Acid detergent fiber (ADF)	21.8	21.8	23.3		
Starch	19.0	15.8	11.2		
Crude fat	3.99	4.98	7.16		
**Volume and composition of dairy slurry, kg·day^−1^·cow^−1^**					
Slurry	76.1^b^	80.2^ab^	84.4^a^	2.55	<0.01
Feces	51.9^b^	55.2^b^	59.8^a^	2.33	<0.01
Urine	24.3^a^	25.0^a^	24.6^a^	0.34	0.71
Dry matter ^β^	6.85^b^	7.28^b^	8.06^a^	0.228	<0.01
Volatile solids	5.98^b^	6.39^b^	7.05^a^	0.203	<0.01
Nitrogen	0.395^b^	0.406^ab^	0.426^a^	0.0125	0.05
Fat	0.433^c^	0.557^b^	0.737^a^	0.0231	<0.01
Neutral detergent fiber (NDF)	3.30^b^	3.55^b^	4.30^a^	0.131	<0.01
ADF	2.00^b^	2.09^b^	2.30^a^	0.081	<0.01
Hemicellulose	1.31^b^	1.46^b^	2.00^a^	0.067	<0.01

^α^ SEM: Standard Error of the Mean; *p*-Value for diet effect. Means on the same line with different superscripts (^a,b,c^) differ significantly (*p* < 0.05); ^β^ DM: dry matter; NDF; ADF; hemicellulose. The number of samples for the compositional analysis of the dairy slurry was 36 from all testing periods.

### 2.2. Incubation Set-Up

**Figure 1:**
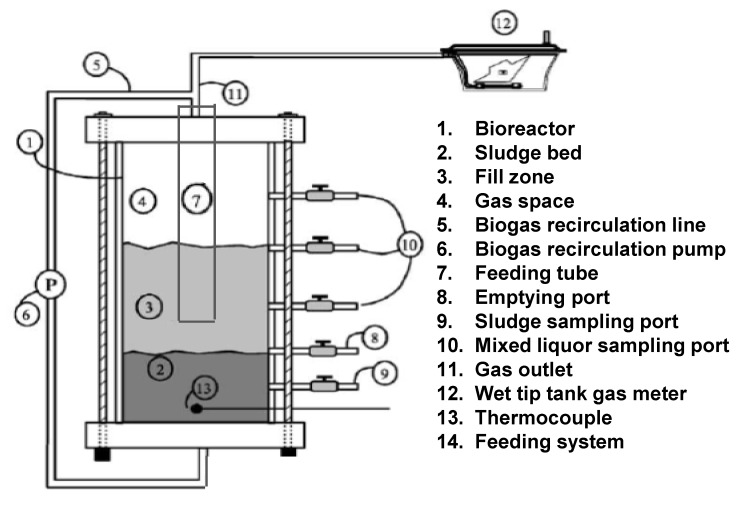
Schematic diagram of the sequence batch reactor.

Bioenergy production measurements were performed on laboratory-scale anaerobic sequencing batch reactors (SBRs) operated under psychrophilic conditions. Six 54-L SBRs located in a controlled-environmental chamber operated at 25 ± 1 °C were used over a 4-month period. The details of the SBR are shown in Figure 1. At the beginning of the experiment, each SBR contained 20 kg of psychrophilic anaerobic inoculum obtained from a semi-commercial scale SBR operated at a temperature of 25 °C. The physico-chemical characteristics of the inoculum are given in Table 2. The semi-industrial digester was located at the Dairy and Swine Research and Development Centre, Sherbrooke, Quebec-Canada. The hydraulic retention time (HRT) was 30 d; the SBRs were operated with feed and react periods of 2 weeks each. The first 3-month period of operation was used to ensure that a steady state was reached and the last month, to determine daily biogas production. The organic loading rate (OLR) was equivalent to 3 g of COD L^−1^·day^−1^ during the feeding period. The amounts of slurry fed into the SBRs were 6.00 kg for the DDGS0 diet; 6.72 kg for the DDGS10 diet; and 6.00 kg for the DDGS30 diet. The contents of the SBRs were mixed twice a week for 5 min. A 100-mL sample of mixed liquor was collected after 5 min of mixing and analyzed to assess the level of degradation of the organic matter. Daily biogas production from slurry corresponding to each diet treatment (2 SBRs/dietary treatments) was monitored. 

**Table 2:** The physico-chemical characteristics of the inoculum.

Parameter	Concentration
Volatile fatty acids (VFAs)	48.1 mg·L^−1^
pH	7.48
Dry matter (DM)	3.4%
Volatile solids (VS)	2.4%
Fixed solids (FS)	1.0%
Soluble chemical oxygen demand (SCOD)	6.8 g·L^−1^
Total chemical oxygen demand (TCOD)	37.3 g·L^−1^
N-ammonia (N-NH_3_)	1.8 g·L^−1^
Total Kjeldahl nitrogen (TKN)	2.8 g·L^−1^

### 2.3. Monitoring Gaseous Emissions

All storage structures and SBRs were closed hermetically in order to capture and measure daily biogas production with wet tip gas meters. Biogas samples were collected once a week using a plastic syringe and analyzed to determine the proportion (in %) of CH_4_ in the biogas with Hach Carle 400 AGC gas chromatograph (Model 04131-C, Hach, Loveland, CO, USA) configured for the application 131-C. The application uses a column (1/8 inches) composed of 1.8 m (805 porapak N + 205 Porapak Q), 2.1 m (80% molecular sieve 13X + 20% molecular Sieve 5A), and 1.8 m (80% OV-101 on chromosorb WHP). The column and thermal conductivity detector were operated at 85 °C with a helium gas flow rate of 30 mL·min^−1^ [7]. Calibration was performed weekly with a standard gas (27.3% CO_2_, 1.01% N_2_, 71.69% CH_4_, 0.53% H_2_S). 

Methane (CH_4_) production is reported in normalized litres (_N_l CH_4_), *i.e.*, the CH_4_ volume produced was corrected to standard temperature and pressure (STP) (273 °K; 1 atm) using Equation (1). 


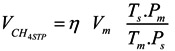
(1)
where *V_m_* is the measured volume of biogas, *η* is the percentage of CH_4_ in biogas, *Tm* and *P_m_* which are the actual temperature and atmospheric pressure at the time of measurement, and *T_s_* and *P_s_* are the standard temperature and pressure. *V*_*CH*_4*STP*__ is the volume of methane at the standard temperature and atmospheric pressure. The yield of biogas and CH_4_ were expressed in _N_L CH_4_·kg^−1^ VS and in _N_L CH_4_ day^−1^·cow^−1^.

### 2.4. Chemical Analyses

Slurries were sampled and analyzed prior each feeding over the 4-month trial and the bioreactor mixed liquors were sampled twice a week during the last 30 d (HRT) and analyzed for pH, DM, VS, FS, Soluble and total chemical oxygen demand (SCOD and TCOD, respectively) [16], phosphorus (P), N-NH_3_,TKN and VFAs. The VFAs (acetic, propionic, butyric, isobutyric, isovaleric) were analyzed using Perkin Elmer gas chromatograph (GC) model 8310 (Perkin Elmer, Norwalk, CT, USA) fitted with FID and, equipped with a J&W Scientific DB-FFAP high resolution column (30 m × 0.53 mm × 1.00 μm; Chromatographic Specialties Inc, Ontario) [7]. The SCOD and TCOD were determined by the closed reflux colorimetric method [16]. The SCOD was measured on the supernatant of a centrifuged sample. The DM content was determined by drying a 10 g subsample for 24 h at 105 °C. Dried solids were then incinerated for 2 h at 550 °C for volatile content measurement. The P and TKN were analyzed on a subsample digested at 420 °C with selenious acid. Concentrations of TKN and N-NH_3_ were determined with a Kjeltec 2400 analyzer (Tecator AB, Hoganas, Sweden). The P concentration was measured with a Lambda 35 spectrometer (Perkin Elmer, Shelton, CT, USA). Cell wall fractions (neutral detergent fiber [NDF] and acid detergent fiber [ADF]) in manure were determined according to the methods of Van Soest and Wine [17] by using a sequential procedure with amylolytic (thermamyl 120 L) treatment.

### 2.5. Calculations and Statistical Analysis

Data on the volume and composition of the three slurries from the study involving 12 dairy cows over the diet testing periods [15] were analyzed with diet as the main effect in a 4 × 4 Latin Square design using the SAS MIXED procedure (SAS release 9.1; SAS Institute Inc., Cary, NC, USA). The composition of the slurries at the beginning and end of the bioenergy trial were analyzed by ANOVA as a completely randomized design with dietary treatment as the main effect, using the SAS MIXED procedure (SAS release 9.1; SAS Institute Inc., Cary, NC, USA). 

CH_4_ production results from the SBRs were also analyzed by ANOVA using the SAS MIXED procedure with diet as the main effect. Multiple means testing was performed using the Tukey test.

The bioenergy production potential was determined by the following equations:

Daily CH_4_ production:

*VCH*_4_(*n*) = *V*(*n*) *VCH*_4_(*n*) (2)

Cumulative CH_4_ over the bioenergy production trial:

*VCH*_4_ = Σ *VCH*_4_(*n*) (3)

Cumulative specific CH_4_ over bioenergy production trial:

*SpecialVCH*_4_ = *VCH*_4_*cumulated / MVS added* (4)

where *n* is the day the measurement is recorded, *V (n)* is the volume (L) of biogas and *CH_4_(n)* is the CH_4_ content in biogas on that specific day, and *MVS* is the total mass of volatile solids fed to the bioreactors (kg). Cumulative *V CH_4_* was obtained by summing the daily CH_4_ production over the one-month trial for bioenergy production. 

To express the bioenergy production in _N_L CH_4_ day^−1^·cow^−1^, the cumulative CH_4_ emissions expressed in _N_L CH_4_ kg^−1^ of VS were divided by the number of days of the trial and multiplied by the amount of VS excreted daily per cow for each dietary treatment. 

## 3. Results and Discussion

### 3.1. Effect of Adding Different Levels of Corn DDGS to the Dairy Cow Diet on Slurry Characteristics

The inclusion of corn DDGS had no effect on urine production but dairy cows fed DDGS30 showed a significant increase in the amount of fresh feces and slurry excreted per day, specifically 15% (*p* < 0.01) and 11% (*p* < 0.01), respectively, compared to the control diet. The different levels of corn DDGS included in the cow diet had a significant influence (*p* < 0.05) on some of the physico-chemical characteristics of manure slurry (Table 1). The inclusion of 10% corn DDGS was associated with a significant increase of 29% (*p* < 0.01) in the daily amount of fat excreted in slurry compared to the control diet. The addition of 30% corn DDGS to the diet caused the following significant increases relative to the control diet: 18% increase in the daily amount of DM (*p* < 0.01); 18% increase in VS (*p* < 0.01); 70% increase in fat (*p* < 0.01); 30% increase in NDF (*p* < 0.01); 15% increase in ADF (*p* < 0.01); and 53% increase in hemicellulose (*p* < 0.01).

There are three possible explanations for the increase in the daily amount of slurry: (i) an increase in DM intake tends to decrease nutrient digestion efficiency because feed passes through the digestive system faster; (ii) an increase in water intake moves digestate faster [18]; and (iii) a high degree of lignification of the fiber in the corn DDGS would make it largely inaccessible for degradation by microorganisms present in the digestive tract [19]. Weiss and Wyatt [20] reported that manure output usually increases as the concentration of dietary fiber (NDF) increases. This occurs because NDF is generally less digestible than other nutrients. On average, a 1% unit increase in NDF concentration increases manure output by 0.23 to 0.45 kg·day^−1^. A decrease in fiber digestibility could also explain the increase in the daily amount of DM, VS, NDF, ADF and hemicellulose excreted in connection with the diet including 30% corn DDGS. 

### 3.2. Effects of Diets on Bioenergy Production

The initial composition of the slurries fed into the SBRs at the beginning of the 4-month trial showed that the slurries resulting from corn DDGS diets differed significantly from the control with respect to certain physico-chemical characteristics, specifically DM, TVFA, pH, TCOD, SCOD, TKN and NH_3_ (Table 3). In the case of the slurry from the DDGS10 diet, the TVFA and SCOD values were significantly higher (18% and 9%, respectively) and the pH, TKN and NH_3_ contents were significantly lower (1%, 4% and 5%, respectively) than for the control diet. The DM, TVFA, SCOD, TKN and NH_3_ in the slurry from the DDGS30 diet were significantly lower (6%, 22%, 7%, 4% and 5%, respectively) and TCOD was significantly higher (9%) than for the control diet (Table 3). 

**Table 3:** Initial and final composition of slurry and CH_4_ production during the bioenergy production trial as a function of feeding strategy (inclusion of 0, 10%, 30% corn dried distillers grains with solubles – DDGS0, DDGS10 and DDGS30)

	DDGS0	DDGS10	DDGS30	SEM^α^	*p*-Value
**Initial composition,% DM**					
Dry matter (DM, %)	6.18^a^	6.13^a^	5.84^b^	0.04	<0.05
Volatile solids (VS)	74^a^	74^a^	75^a^	0.20	0.12
Fixed solids (FS)	26^a^	26^a^	25^a^	0.20	0.12
Total volatile fatty acids (TVFA)	5.28^b^	6.21^a^	4.12^c^	0.04	<0.01
pH	7.40^a^	7.32^b^	7.36^ab^	0.008	<0.05
Total chemical oxygen demand (TCOD)	109^b^	109^b^	119^a^	1.75	<0.05
Soluble COD	26.8^b^	29.2^a^	24.8^c^	0.28	<0.01
Total Kjedahl nitrogen (TKN)	7.57^a^	7.31^b^	7.20^b^	0.03	<0.01
Ammonia (NH_3_)	5.28^a^	5.01^b^	4.98^b^	0.03	<0.01
**Final composition,% DM**					
DM (%)	5.41^a^	5.40^a^	4.79^a^	0.15	0.09
VS	70^a^	71^a^	69^a^	0.71	0.20
FS	30^a^	29^a^	31^a^	0.71	0.20
TVFA	0.29^a^	0.36^a^	0.40^a^	0.03	0.14
pH	7.62^a^	7.59^a^	7.59^a^	0.01	0.28
TCOD	106^ab^	108^a^	103^b^	0.48	<0.05
SCOD	23.5^a^	22.7^a^	19.2^b^	0.47	<0.05
TKN	8.70^a^	8.30^a^	8.32^a^	0.52	0.84
NH_3_	6.60^a^	6.12^a^	6.53^a^	0.22	0.38
**Bioenergy production**					
_N_L CH_4_ kg^−1^ VS	255.8^a^	265.0^a^	252.9^a^	1.58	0.34
_N_L CH_4 _day^−1^·cow^−1^	947.5^b^	1054^ab^	1084^a^	13.23	<0.05

^α ^SEM: Standard Error of the Mean; P-Value for diet effect. Means on the same line with different superscript letters (^a,b,c^) differ significantly (*p* < 0.05). The data in the table is the average of 8 samples for each diet.

The final composition indicated that residual TVFA were nearly zero for all dietary treatments (Table 3) and that SCOD was significantly lower (18%) in the slurry from the DDGS30 diet than in the control diet. 

Bioenergy production expressed in _N_L CH_4_·kg^−1^ VS was not affected significantly by diet. However, when the increase in the volume of manure excreted daily per cow with the addition of corn DGGS is considered, it can be seen that daily CH_4_ production per cow increased significantly in the slurry from the DDGS30 diet (14%) compared to the control diet (Table 3). These results can be explained by the fact that the amount of slurry excreted daily per cow was significantly higher for the DDGS30 diet than for the other two diets. Similar findings have been reported for incorporating different fiber contents in pig diets [14] and for incorporating fat in cattle diets [21]. Based on what is reported in the literature, the higher bioenergy production from the DDGS30 treatment could be expected because the organic content of this slurry was greater than that of the slurries from the other two diets. It appears that the increase in bioenergy production is not attributable solely to the amount of nutrients excreted and that the type of nutrients had an effect. This study shows that the inclusion of 30% DDGS caused a significant increase in the amount of fats, NDF, ADF and hemicellulose (0.304, 1.0, 0.30, and 0.69 kg d^−1^·cow^−1^, respectively (Table 1)) excreted in the slurry. Fibers such as lignin are known to build complexes with cellulose and hemicellulose, thus creating a physical barrier making it difficult for microbial enzymes to break down the organic compounds in short time. However, 80% degradation of carbohydrate fibers requires around 30 days [22] which has been provided by the HRT used in this study. Notice that the theoretical methane potential per gram of VS is significantly greater for fat compared to proteins and carbohydrate (1014, 496, and 415 _N_L CH_4_·kg^−1^ VS, respectively) [22].

## 4. Conclusion

Although the inclusion of corn DDGS had no effect on urine production, dairy cows fed DDGS30 showed a significant increase of 11% and 15% in the amount of fresh slurry and fresh feces excreted per day, respectively. The DDGS10 treatment caused a significant increase (29%) in the daily amount of fat, and the DDGS30 treatment increased the daily DM, volatile solids (VS), fat, neutral detergent fiber (NDF), acid detergent fiber (ADF) and hemicellulose contents by 18%, 18%, 70%, 30%, 15% and 53%, respectively, compared to the control diet. The bioenergy production trial showed that the inclusion of corn DDGS30 caused a significant increase of 14% in daily bioenergy production (_N_L CH_4 _day^−1^·cow^−1^). These data will be used to carry out a detailed and comprehensive life cycle analysis to assess the level of reduction in carbon footprint of Canadian dairy products via the adoption of anaerobic digestion and inclusion of DDGS in dairy diets.
